# Editorial: Insights in terrestrial microbiology: 2022

**DOI:** 10.3389/fmicb.2023.1347778

**Published:** 2024-01-05

**Authors:** Luis Raul Comolli, Kim Yrjälä

**Affiliations:** ^1^Independent Researcher, Basel, Switzerland; ^2^Department of Forest Sciences, University of Helsinki, Helsinki, Finland

**Keywords:** phytoremediation, organic amendments, microbial communities, ecopiling, soil organic matter (SOM), bacterial succession

Soil health is intrinsically connected to environmental sustainability and merges soil microbes with plants in carbon management. It is of fundamental importance to develop better soil management practices that significantly improve carbon fixation, enhancing soil fertility while avoiding increases in greenhouse gas emissions. The focus is on maintaining a balance between productive agricultural practices and environmental sustainability that includes well-planned field studies. Beyond agriculture, phytoremediation is a promising solution for the remediation of hydrocarbon- and heavy metal contaminated soils, which is a prevalent issue associated with industrial pollution, but also natural intrinsic soil conditions and paves the way for safer crop production.

Soil salinization seriously restricts the development of agricultural production. Globally, 25% of the total land is affected by salinity, an area that is growing at a rate of more than 1 million ha per year. Microbial communities in high-saline environments allocate resources toward survival and resource acquisition at the expense of growth yield (Ning et al.), thereby impeding SOC formation. The question is, then, what can be done to increase the fertility of these salt affected soil? One approach is to use plants in phytoremediation to improve the quality of the soils (Lopez-Echartea et al., 2019). Phytoremediation can be combined with organic amendments of agricultural residues to improve the quality of the saline and sodic soil (Miranda et al., 2021). The work reported that the treatments with the plant *A. nummularia* and sheep manure application decreased soil electric conductivity values below the boundary that classifies saline soils (4.0 dS m^−1^). These studies combined point to the importance of studying phytoremediation complemented with the use of organic conditioners produced on the farm, for sustainable soil management.

The use of straw uncovered a positive correlation between microbial CUE and the proportion of heterocyclic compounds in the amendments (Li et al.). This effect was promoted by fungal dominance in the microbial community, which in turn enhances the microbial use efficiency of straw carbon in agricultural ecosystems. Different amounts of straw mulch had a considerable impact on the microbial beta diversity and promoted processes essential for soil carbon and nitrogen cycles. The straw treatment also shifted microbial communities toward fungi, which could be instrumental in enhancing the production of labile carbon and nitrogen, accelerating the cycles of these nutrients in maize fields. Liu et al. (2020) used two different soils and compared CUE of the straw amendment with biochar amendment in 160-day The results showed the biochar amendment increased the carbon use efficiency in both studied soils compared to straw amendment due to increased portion of fungi. The use of straw mulch decreased aromatic carbon degradation, fermentation, and nitrate reduction in another study (Wu et al., 2019), which would imply putative decrease in greenhouse gasses (GHG). This work assessed the impacts of straw addition in agricultural soil subjected to long-term mineral and organic fertilization. They found that it is important to manage straw incorporation in such way that it increases straw-derived SOC and alleviate existing SOC mineralization, to optimize the C sequestration efficiency.

Different thinning intensities in a plantation of *Cryptomeria japonica var. sinensis*, a significant timber species in China, was studied by Liu et al.. This study contributes to the understanding of how thinning strategies can be developed to balance ecological and economic benefits which hold considerable importance for the region's economic development and environmental protection. These results can provide the valuable insights for new work across other important timber species worldwide.

Ecopiles treated with phytoremediation and amended with nitrogen fertilization, and local bacteria was successful in reduction of total petroleum hydrocarbons, with a 99% decrease in aliphatics and an 88% decrease in aromatics (Martínez-Cuesta et al.). The microbial community analysis revealed a succession pattern transitioning from hydrocarbon-degrader populations to those typical of clean soils. These results are consistent with previous *ex situ* phytoremediation field study in the Boreal vegetation zone where a distinct succession of microbial communities was revealed during the break down of petroleum hydrocarbon compounds (Mukherjee et al., 2015). The research highlights ecopiling as a cost-effective and successful method for not only degrading hydrocarbons but also for restoring microbial communities to a state representative of clean soils, emphasizing the potential of ecopiling for both soil decontamination and ecological restoration.

Sansupa et al. surveyed the microbiome present in one unpreserved ancient mural in Thailand, to establish the connection to other historical mural paintings, and point out future work needed to halt biodeterioration of these types of work of art in general. Their work follows in the steps of the landmark publication by Rosado et al. (2014) which gave a description of the microbial population that develops on mural paintings, giving a detailed overview of contaminants that was not possible with the other previous techniques. In that publication they gave a description of the microbial population that develops on the mural paintings providing a detailed overview of contaminants that was not possible with previous techniques. The knowledge of the uncultured diversity started to grow in the late 1990′s with the use of molecular genetic methods like DGGE (Galand et al., 2002) and TRFLP (Juottonen et al., 2008). The identification of microbes back then required cloning that reduced the capacity to identify the true diversity of microbes in samples (Mukherjee et al., 2013). Later high-throughput sequencing methods became widely available and were combined with bioinformatic handling of the massive microbial data (Mukherjee et al., 2015). It is possible to reveal the scale of microbial communities in cultural heritage artifacts like mural paintings, but there is still a gap in our capacity to know the functions of the detected microbes. PICRUST (Langille et al., 2013) and Tax4fun (Aßhauer et al., 2015) are bioinformatic algorithms used for prediction of the functional capacity of detected microbial communities that we expect to be more widely used in the future to overcome the microbial functional dilemma.

To conclude microbial ecology studies by up-to-date microbial analysis combined with plant and soil interactions gives opportunities to fill in the gaps concerning how natural or added carbon is circulated in soil and the possibility for carbon sequestration.

## Author contributions

LC: Writing – original draft, Writing – review & editing. KY: Conceptualization, Writing – original draft, Writing – review & editing.
